# Postpartum glucose follow-up after gestational diabetes in China: provider and institutional determinants in a multicenter survey across urban public hospitals

**DOI:** 10.3389/fendo.2026.1887259

**Published:** 2026-08-07

**Authors:** Yinge Wang, Siying Li, Fangying Huang, Jingya Li, Xue Lian, Lihua Huang, Weizhen Wu, Yingtao Li

**Affiliations:** 1 The Third School of Clinical Medicine, Guangzhou Medical University, Guangzhou, Guangdong, China; 2 Department of Obstetrics and Gynecology; Guangdong Provincial Key Laboratory of Major Obstetric Diseases; Guangdong Provincial Clinical Research Center for Obstetrics and Gynecology; Guangdong-Hong Kong-Macao Greater Bay Area Higher Education Joint Laboratory of Maternal-Fetal Medicine; The Third Affiliated Hospital, Guangzhou Medical University, Guangzhou, Guangdong, China

**Keywords:** cross-sectional survey, gestational diabetes mellitus, implementation, obstetric healthcare professionals, postpartum glucose follow-up, type 2 diabetes prevention

## Abstract

**Background:**

Postpartum glucose follow-up after gestational diabetes mellitus (GDM) represents an important opportunity for early diabetes risk identification and long-term type 2 diabetes prevention. Obstetric healthcare professionals play a key role in initiating postpartum follow-up, yet evidence on provider- and institution-related factors influencing implementation in routine obstetric care remains limited.

**Methods:**

A multicenter cross-sectional survey was conducted among 638 obstetric healthcare professionals from 128 participating urban public hospitals across 15 provinces in China. A self-developed questionnaire was refined through two rounds of Delphi expert consultation and evaluated using exploratory factor analysis. Data were analysed using descriptive statistics, Pearson’s correlation analysis, Mann–Whitney U tests, Kruskal–Wallis H tests, and robust regression based on a generalised linear model.

**Results:**

Mean scores for knowledge, professional beliefs, and practice were 4.07 ± 0.57, 3.99 ± 0.40, and 3.89 ± 0.73, respectively, indicating that practice lagged behind knowledge and professional beliefs. Knowledge was positively correlated with professional beliefs (r = 0.473, P < 0.001) and practice (r = 0.630, P < 0.001). Participation in specialised GDM training, experience working in a specialist GDM clinic, and self-reported mastery of relevant knowledge were associated with higher scores across domains. Hospital-level differences were observed for practice scores, but these differences did not indicate a simple gradient by hospital level.

**Conclusion:**

This study suggests that implementation of postpartum glucose follow-up after GDM in routine obstetric care is associated with both provider- and institution-related factors. Although obstetric healthcare professionals generally demonstrated good knowledge and positive professional beliefs, practice remained comparatively weaker. Specialised training, clinical experience, and institutional context were associated with implementation-related practice. Future efforts to strengthen training, optimise follow-up pathways, and improve institutional support may help reduce missed opportunities for postpartum follow-up and strengthen long-term diabetes prevention after GDM.

## 1 Introduction

Gestational diabetes mellitus (GDM) is one of the most common metabolic complications of pregnancy and represents a growing global public health and clinical diabetes concern (1). According to the 11th edition of the International Diabetes Federation Diabetes Atlas, hyperglycaemia in pregnancy affects approximately one in five live births worldwide, and approximately one in six live births are affected specifically by GDM (1). A global analysis using International Association of Diabetes and Pregnancy Study Groups criteria estimated the standardised prevalence of GDM to be 14.0%, with substantial regional variation (2). In addition to its adverse effects on perinatal outcomes for women and infants, GDM is increasingly recognised as an early marker of future metabolic risk (3–5). Women with a history of GDM are at increased risk of type 2 diabetes, obesity, hypertension, and other chronic conditions later in life, and their offspring are also at increased risk of adverse metabolic outcomes (6–8). Meta-analytic evidence has shown that women with prior GDM have an overall relative risk of approximately 9.51 for developing type 2 diabetes compared with those without GDM (8). The estimated absolute risk of type 2 diabetes after GDM also increases over longer postpartum follow-up, reaching 19.72%, 29.36%, 39.00%, 48.64%, and 58.27% at 10, 20, 30, 40, and 50 years postpartum, respectively (9). These findings indicate that GDM is not only a transient pregnancy-related condition but also an important marker of long-term metabolic risk.

Although the intergenerational consequences of GDM are important, the mother’s postpartum metabolic trajectory also requires sustained attention. Timely postpartum glucose follow-up provides a critical opportunity for early diabetes risk identification and secondary prevention of type 2 diabetes, while inadequate postpartum glycaemic management may affect subsequent pregnancies and women’s long-term metabolic health (3, 10, 11).

International clinical guidelines consistently recommend that women with GDM undergo postpartum glucose testing, typically with an oral glucose tolerance test (OGTT) at 4–12 weeks after delivery, and receive professional support for ongoing glucose management and follow-up (3, 4, 10). However, implementation of these recommendations in routine clinical practice remains suboptimal, particularly in relation to women’s completion of guideline-recommended postpartum glucose testing. Previous studies have mainly focused on women’s adherence to postpartum glucose testing after GDM and have consistently reported suboptimal completion of recommended screening (12, 13). For example, a systematic review reported persistent gaps in postpartum follow-up and diabetes screening among women with GDM (13), and a large integrated health system study further showed that completion of the guideline-recommended 2-hour OGTT within 4–12 weeks postpartum remained below optimal levels (12). These findings highlight the importance of women’s adherence, but they also suggest that patient behaviour alone cannot fully explain the implementation gap. Whether women receive clear recommendations, risk counselling, referral support, and continuity of care may also depend on healthcare professionals and institutional follow-up pathways. Therefore, postpartum glucose follow-up after GDM should be understood as both a patient-level adherence issue and a provider- and institution-level implementation issue.

The persistent gap between guideline recommendations and real-world postpartum follow-up is increasingly important as the burden of GDM continues to rise and as health systems seek to prevent type 2 diabetes earlier in the life course. Existing provider-focused studies on postpartum follow-up after GDM, including surveys from Ireland and the United Kingdom, have largely been conducted in high-income settings, whereas evidence from China remains limited (14, 15). This knowledge gap is relevant because obstetric service organisation, postpartum care pathways, and diabetes prevention services may differ substantially across health systems.

A multicenter survey across participating urban public hospitals in China may therefore provide context-specific evidence on this underexplored implementation issue.

In routine obstetric care, obstetric healthcare professionals are often the first, and sometimes the only, professionals who counsel women with GDM before discharge and during early postpartum visits (3, 10, 11). They play a central role in providing risk counselling, recommending postpartum glucose testing, arranging referral, and supporting continuity of care (3, 10, 11). Understanding their knowledge, professional beliefs, and implementation-related practice is therefore important for identifying provider- and institution-level factors that may shape postpartum glucose follow-up after GDM.

The Knowledge–Attitude–Practice (KAP) framework is useful in this context because knowledge alone may be insufficient to ensure implementation; the attitude component, operationalised in this study as professional beliefs, may influence whether knowledge is translated into consistent clinical practice (16, 17). Guided by this framework, this multicenter survey examined obstetric healthcare professionals’ knowledge, professional beliefs, and implementation-related practice regarding postpartum glucose follow-up for women with prior GDM in China.

Specifically, we aimed to assess these three domains, examine the relationships among them, and identify provider-level and institution-related factors associated with implementation-related practice. Particular attention was paid to potentially modifiable provider-level factors, including specialised GDM training, specialist GDM clinic experience, and self-reported knowledge mastery, as well as institution-related characteristics such as hospital level. By identifying these factors, this study seeks to provide evidence to inform targeted training, follow-up pathways, and service improvement strategies for strengthening long-term diabetes prevention after GDM.

## 2 Methods

### 2.1 Study design, setting, and participants

This cross-sectional survey was conducted between January and February 2026 among obstetric healthcare professionals in China. A convenience sampling strategy was used to recruit hospitals and individual participants. Hospitals were identified through the research team’s professional network and collaborating obstetric or GDM-related clinical contacts across different regions of China. Eligible hospitals were invited if they provided obstetric care for women with GDM and had healthcare professionals involved in antenatal or postpartum GDM management. In total, 128 public hospitals located in urban areas across 15 provinces participated in the survey. Hospital level was collected and is reported in **Supplementary Table 2**.

Within each participating hospital, the survey was distributed to eligible obstetric healthcare professionals by designated local coordinators. Eligible participants were registered nurses or physicians who were currently working in obstetric departments or GDM-related clinical services and had experience in caring for women with GDM. Participation was voluntary and anonymous. A total of 725 questionnaires were distributed and returned. After excluding 87 invalid questionnaires, 638 valid questionnaires were included in the final analysis, corresponding to a valid response rate of 88.0%. Because recruitment was conducted through institutional contacts and questionnaire completion was anonymous, non-respondent information was not available; this limitation is further addressed in the Discussion.

The inclusion criteria were as follows:

1. licensed nurses or doctors with valid professional registration in China;
2. at least 3 years of obstetric clinical practice experience;
3. at least 1 year of experience in the management of gestational diabetes mellitus (GDM);
4. willingness to participate after being informed of the purpose of the study.

The exclusion criteria were intern or trainee healthcare professionals, and healthcare professionals who were not on duty during the survey period.

### 2.2 Sample size determination

According to Kendall’s rule of thumb for questionnaire-based exploratory factor analysis, the recommended sample size is 5–10 times the number of questionnaire items (18, 19). As the questionnaire comprised 41 items, the minimum required sample size was estimated to be 205–410 participants. After allowing for a potential 20% invalid response rate, the minimum required sample size threshold was set at 492 participants. This threshold was used as the minimum required sample size rather than as a stopping rule for recruitment. Data collection was conducted during the predefined survey period from January to February 2026, and all eligible valid responses obtained during this period were included in the analysis. A total of 725 questionnaires were distributed, all of which were returned; after excluding 87 invalid questionnaires, 638 valid questionnaires were ultimately included.

Kendall’s rule is an empirical guideline for questionnaire validation and exploratory factor analysis rather than a formal hypothesis-testing power calculation. Therefore, a supplementary sensitivity power analysis was conducted using G*Power 3.1 based on the final sample size and standard power-analysis procedures (20). For correlation analyses, the sensitivity test was based on Fisher’s z transformation for Pearson’s correlation. For representative two-group comparisons, sensitivity analyses were conducted using two-tailed independent-samples t tests, with Cohen’s d as the standardised effect size. With a two-sided α level of 0.05 and 80% power, the final sample size of 638 was sufficient to detect a Pearson correlation coefficient of approximately r = 0.11 or greater. For representative two-group comparisons, the available group sizes were sufficient to detect small-to-moderate standardised mean differences, including d = 0.25 for specialised GDM training, d = 0.23 for specialist GDM clinic experience, and d = 0.32 for self-reported knowledge mastery.

### 2.3 Instrument development

The questionnaire was revised through two rounds of Delphi expert consultation. A total of 17 experts in the field of obstetrics were invited to participate in the review. The experts provided suggestions for modification based on the relevance, scientific rigour, and comprehensibility of each item. Following discussion, items considered inappropriate were deleted or revised, and the final questionnaire was established.

The final questionnaire comprised three dimensions with a total of 41 items: 16 knowledge items, 10 professional belief items, and 15 practice items.

#### 2.3.1 General information questionnaire

The general information questionnaire collected data on participants’ sex, age, ethnicity, parental status, hospital name and level, profession, professional title, years of experience in the management of women with GDM, educational level, whether they had received specialised training in GDM management, whether they had worked in a specialist GDM clinic, and whether they considered themselves to have mastered knowledge related to postpartum glucose follow-up after GDM.

#### 2.3.2 Knowledge, professional beliefs, and practice questionnaire on postpartum glucose follow-up after GDM

The questionnaire included three dimensions: knowledge, professional beliefs, and practice.

The knowledge dimension comprised 16 items addressing knowledge related to postpartum glucose follow-up after GDM, including weight control, nutritional management, exercise guidance, medication use, breastfeeding, glycaemic targets, and considerations for subsequent pregnancies.

The professional belief dimension comprised 10 items and mainly reflected clinicians’ perceptions of the importance of postpartum glucose follow-up after GDM, their willingness to undertake such management, and their views on workload and policy support.

The practice dimension comprised 15 items and assessed clinicians’ relevant behaviours in clinical practice, including guideline learning, risk education, screening and follow-up, tracking OGTT results, psychological assessment, and peer support.

All items were rated using a five-point Likert scale, with higher scores indicating higher levels within the corresponding dimension.

#### 2.3.3 Reliability and validity of the questionnaire

The content validity of the questionnaire was evaluated using expert assessment (21). After the second round of expert consultation, the item-level content validity index (I-CVI) for all items was ≥ 0.78, the median score was ≥ 3, and the interquartile range was ≤ 1(Details are presented in **Supplementary Table 1**). The Friedman test showed that Kendall’s *W* was 0.149 (χ² = 101.043, df = 40, *P* < 0.001), indicating statistically consistent expert opinions.

The reliability and structural validity of the questionnaire were examined in the formal survey sample (n = 638). The overall Cronbach’s α coefficient was 0.965, and the α coefficients for the knowledge, professional belief, and practice dimensions were 0.965, 0.865, and 0.944, respectively, indicating good internal consistency (22). The reliability analysis was rechecked, and the identical values for the overall scale and the knowledge dimension were confirmed. The high internal consistency of the knowledge dimension may be explained by the fact that the knowledge items were measured using a five-point Likert scale and were designed to assess a coherent self-reported knowledge domain related to postpartum glucose follow-up after GDM.

Exploratory factor analysis showed a Kaiser–Meyer–Olkin value of 0.964, and Bartlett’s test of sphericity was significant (χ² = 23446.374, *P* < 0.001), indicating that the data were suitable for factor analysis (18, 19). Principal component analysis with varimax rotation extracted four factors with eigenvalues greater than 1, accounting for 66.86% of the total variance (18, 19). Inspection of the rotated component matrix showed that Factor 1 consisted of all knowledge items (K1–K16; primary loadings 0.628–0.812), Factor 2 consisted mainly of practice items (P1–P15; primary loadings 0.505–0.793) together with one professional belief item assessing perceived capability to independently perform postpartum glucose management and follow-up (A7; primary loading 0.465), Factor 3 consisted of positive professional belief items (A1–A6 and A8; primary loadings 0.690–0.877), and Factor 4 consisted of two negatively worded professional belief items reflecting perceived workload and policy-support barriers (A9–A10; primary loadings 0.824–0.826). Therefore, the four-factor solution largely corresponded to the original KAP framework, although the professional belief domain separated into positive professional beliefs and perceived implementation barriers, and A7 loaded with the practice factor because of its capability-related content. The original three-domain scoring structure was retained for subsequent analyses because it was specified *a priori* based on the KAP framework and Delphi consultation. No items were deleted after EFA. Substantial cross-loading was defined as a secondary factor loading ≥0.40. One item, P9, which assessed breastfeeding education, showed cross-loading on the positive professional belief factor, with a primary loading of 0.578 on the practice factor and a secondary loading of 0.455 on the positive professional belief factor. This item was retained because its primary loading was higher and its content was theoretically consistent with the practice domain. The rotated factor loading summary is presented in **Supplementary Table 5**.

### 2.4 Data collection

Data were collected using an electronic questionnaire. The questionnaire link and QR code were generated through the Wenjuanxing platform and distributed to participants. The questionnaire was completed anonymously, all items were mandatory, and each device was permitted to submit the questionnaire only once.

After questionnaire collection, two researchers reviewed the data for quality and excluded questionnaires with obvious logical errors or patterned responses.

### 2.5 Ethics approval and consent to participate

The Declaration of Helsinki was followed in the conduct of the research. This study was approved by the Clinical Research and Application Ethics Committee of the Third Affiliated Hospital of Guangzhou Medical University (permission number: Linlun Review (IIT) (2025) No.122). All participants took part voluntarily on the basis of informed consent. The questionnaire was completed anonymously, and all information was used solely for scientific research and kept strictly confidential.

### 2.6 Statistical analysis

Data entry was performed by two members of the research team.

Data were analysed using SPSS version 27.0. Continuous variables were presented as mean ± standard deviation, with 95% CI and distributional characteristics also reported.

Pearson’s correlation analysis was used to examine associations among the main variables. Group comparisons were conducted using the Mann–Whitney *U* test and Kruskal–Wallis *H* test, with Bonferroni *post hoc* correction applied.

Between-group comparisons using the Mann–Whitney U test and Kruskal–Wallis H test were conducted as preliminary univariable analyses to describe differences in KAP scores across participant and institutional characteristics. Multivariable generalised linear models were then constructed for the knowledge, professional belief, and practice scores. Because residual diagnostics indicated heteroscedasticity, the models were fitted using a normal distribution and identity link function, with robust standard errors estimated using a robust covariance matrix. The full multivariable models included prespecified candidate variables based on theoretical relevance and prior evidence, including sex, age, parental status, collapsed hospital level, profession, professional title, years of experience in managing women with GDM, highest educational qualification, participation in specialised GDM training, experience working in a specialist GDM clinic, and self-reported mastery of knowledge related to postpartum glucose management after GDM.

Categorical variables were entered as indicator variables, with one category specified as the reference group for each variable. Reference groups are reported in the regression tables. Multicollinearity diagnostics were conducted for the predictors included in the multivariable models. Because the predictors were categorical, they were dummy-coded for diagnostic purposes, with stable reference categories selected where appropriate. Variance inflation factors (VIFs), tolerance values, and condition indices were examined.

In addition, parsimonious models focusing on modifiable provider-level factors were constructed for specialised GDM training, specialist GDM clinic experience, and self-reported knowledge mastery. Statistical significance was set at *P* < 0.05.

As healthcare professionals were recruited from 128 hospitals across 15 provinces, additional sensitivity analyses were conducted to assess the potential influence of clustering. Because specific hospital identifiers could not be reliably defined in the de-identified analytic dataset, hospital-level random intercepts could not be specified. Therefore, linear mixed-effects models with province as a random intercept were fitted as sensitivity analyses. Intraclass correlation coefficients (ICCs) were estimated from unconditional random-intercept models for the knowledge, professional belief, and practice scores. The same prespecified fixed-effect variables used in the full multivariable models were then included in the mixed-effects models, and the results were compared with those from the robust generalised linear models.

This study was reported in accordance with the STROBE statement for cross-sectional studies.

Because convenience sampling was used, no sampling weights were applied in the analysis. Because only two participants were recruited from secondary level-B hospitals, secondary level-A and secondary level-B hospitals were combined into a single “secondary hospitals” category before the primary multivariable analyses to improve the stability of hospital-level estimates. Hospital level was therefore analysed as three categories: secondary hospitals, tertiary level-B hospitals, and tertiary level-A hospitals, with tertiary level-A hospitals used as the reference category in the regression models.

## 3 Results

### 3.1 General characteristics of the participants

A total of 725 questionnaires were distributed to obstetric healthcare professionals from 128 public hospitals located in urban areas across 15 provinces in China. All 725 questionnaires were returned. After excluding 87 invalid questionnaires, 638 valid questionnaires were included in the final analysis, yielding a valid response rate of 88.0%. Because all questionnaire items were mandatory, no missing data were identified (details are presented in **Supplementary Table 2**).

### 3.2 Knowledge, professional beliefs, and practice scores related to postpartum glucose follow-up after GDM among obstetric healthcare professionals

A total of 638 valid questionnaires were included in this study. The mean score for the knowledge dimension was 4.07 ± 0.57 (95% CI 4.05–4.10), the mean score for the professional belief dimension was 3.99 ± 0.40 (95% CI 3.97–4.01), and the mean score for the practice dimension was 3.89 ± 0.73 (95% CI 3.86–3.92). Details are shown in **Table 1**.

**Table 1:** Knowledge, professional beliefs, and practice of obstetric healthcare professionals regarding postpartum glucose follow-up after GDM.

Dimension	N	Mean ± SD	95% CI	Median	Min–Max	Skewness	Kurtosis
Knowledge	638	4.07 ± 0.57	4.05–4.10	4.00	1.19–5.00	-0.43	1.98
Professional beliefs	638	3.99 ± 0.40	3.97–4.01	4.00	2.70–5.00	0.15	-0.11
Practice	638	3.89 ± 0.73	3.86–3.92	3.93	1.13–5.00	-0.56	0.73

### 3.3 Item-level analysis of knowledge, professional beliefs, and practice regarding postpartum glucose follow-up after GDM

The overall mean score for the knowledge dimension was 4.07 ± 0.57. The highest agreement rates were observed for “understanding postnatal glycaemic targets” (94.20%) and “understanding the principles of diagnosis and management of hypoglycaemia” (94.04%). Lower agreement rates were observed for “understanding guidance and tracking using digital health technologies” (72.88%) and “understanding the latest research evidence and clinical guidelines” (76.96%).

In the professional belief dimension, agreement exceeded 96% for all positively worded items. For negatively worded items, 76.65% of participants agreed that “postnatal glucose management increases workload”, and 71.32% agreed that “policy support is insufficient”.

In the practice dimension, the highest reported rate was for breastfeeding education (89.03%). However, only 49.84% of participants reported that they had actually implemented postpartum glucose follow-up for women with GDM. The proportions reporting long-term follow-up tracking and standardised process documentation were 57.21% and 64.11%, respectively. Details are presented in **Supplementary Table 3**.

### 3.4 Correlation analysis of knowledge, professional beliefs, and practice scores

Pearson’s correlation analysis showed that knowledge, professional belief, and practice scores were all positively correlated (*P* < 0.001). Knowledge was positively correlated with professional beliefs (*r* = 0.473, 95% CI 0.410–0.531) and practice (*r* = 0.630, 95% CI 0.581–0.675). Professional beliefs were also positively correlated with practice (*r* = 0.473, 95% CI 0.410–0.531). Details are shown in **Table 2**.

**Table 2:** Pearson’s correlation analysis of knowledge, professional belief, and practice scores (*n* = 638).

Variable	Knowledge score	Professional belief score	Practice score
Knowledge score	1	0.473 (95% CI 0.410–0.531)***	0.630 (95% CI 0.581–0.675)***
Professional belief score	0.473 (95% CI 0.410–0.531)***	1	0.473 (95% CI 0.410–0.531)***
Practice score	0.630 (95% CI 0.581–0.675)***	0.473 (95% CI 0.410–0.531)***	1

***P < 0.001.

### 3.5 Between-group differences in knowledge, professional belief, and practice scores according to participant characteristics

The Mann–Whitney *U* test showed significant differences in knowledge scores according to participation in specialised training, experience working in a specialist GDM clinic, and self-reported mastery of relevant knowledge (all *P* < 0.001). Knowledge scores were higher among participants who had received specialised training than among those who had not (*Z* = -8.497, *P* < 0.001), higher among those with specialist GDM clinic experience than among those without such experience (*Z* = -6.045, *P* < 0.001), and higher among those reporting mastery of relevant knowledge than among those who did not (*Z* = -9.350, *P* < 0.001). No statistically significant differences in knowledge scores were found according to sex, parental status, or profession (*P* > 0.05).

In the professional belief dimension, participants who had received specialised training and those who reported mastery of relevant knowledge had higher scores than their comparison groups (both *P* < 0.001). No statistically significant differences were observed for the remaining variables.

In the practice dimension, participants who had received specialised training, had specialist GDM clinic experience, and reported mastery of relevant knowledge had higher scores (all *P* < 0.001). No statistically significant differences were found according to sex, parental status, or profession.

After secondary level-A and secondary level-B hospitals were combined into a single secondary-hospital category, the Kruskal–Wallis H test showed significant differences in knowledge scores across hospital levels (χ² = 8.236, df = 2, P = 0.016). *Post hoc* pairwise comparisons with Bonferroni correction showed that participants working in tertiary level-B hospitals had higher knowledge scores than those working in secondary hospitals (adjusted P = 0.012), whereas the other pairwise comparisons were not statistically significant. Significant differences were also observed in practice scores across hospital levels (χ² = 12.862, df = 2, P = 0.002). *Post hoc* pairwise comparisons showed that participants working in tertiary level-B hospitals had higher practice scores than those working in secondary hospitals (adjusted P = 0.003) and tertiary level-A hospitals (adjusted P = 0.001). No significant difference in practice scores was observed between secondary hospitals and tertiary level-A hospitals. No significant differences were observed in professional belief scores across hospital levels (χ² = 2.935, df = 2, P = 0.230).

Significant differences were also observed according to years of work experience in the knowledge dimension (χ² = 10.292, df = 4, P = 0.036) and practice dimension (χ² = 10.319, df = 4, P = 0.035). *Post hoc* comparisons showed that clinicians with 6–10 years of work experience had higher knowledge scores than those with ≤5 years of work experience, and clinicians with 16–20 years of work experience had higher practice scores than those with ≤5 years of work experience (adjusted P < 0.05). Professional belief scores did not differ significantly according to years of work experience (χ² = 5.597, df = 4, P = 0.231). Details are presented in **Supplementary Table 4**.

### 3.6 Multivariable robust regression analysis

Robust regression analysis was performed using generalised linear models with a normal distribution and identity link function, with robust standard errors estimated using a robust covariance matrix. Multicollinearity diagnostics did not indicate severe multicollinearity among the predictors. The VIF values ranged from 1.034 to 4.888, tolerance values ranged from 0.205 to 0.967, and the maximum condition index was 20.587. Details are presented in **Supplementary Table 7**.

**Tables 3**–**5** present the full multivariable models including prespecified participant- and institution-related variables. **Table 6** presents parsimonious models focusing on the three potentially modifiable provider-level factors: specialised GDM training, specialist GDM clinic experience, and self-reported knowledge mastery.

**Table 3:** Robust regression analysis of factors associated with knowledge scores.

Independent variable	Reference group	*B*	SE	Wald χ²	df	*P*
Sex (male)	Female	0.050	0.091	0.303	1	0.582
Age <30 years	≥40 years	0.014	0.098	0.021	1	0.884
Age 30–39 years	≥40 years	-0.016	0.061	0.071	1	0.789
Parental status (yes)	No	0.003	0.068	0.002	1	0.964
Hospital level: secondary hospitals	Tertiary level-A hospitals	-0.059	0.050	1.368	1	0.242
Hospital level: tertiary level-B hospitals	Tertiary level-A hospitals	0.145	0.079	3.340	1	0.068
Profession (nurse)	Doctor	0.060	0.057	1.102	1	0.294
Professional title (senior)	Other	0.004	0.123	0.001	1	0.974
Professional title (associate senior)	Other	-0.040	0.081	0.247	1	0.619
Professional title (intermediate)	Other	-0.064	0.058	1.214	1	0.270
Years of experience (≤5 years)	≥20 years	-0.147	0.091	2.647	1	0.104
Years of experience (6–10 years)	≥20 years	-0.024	0.089	0.070	1	0.791
Years of experience (11–15 years)	≥20 years	-0.053	0.090	0.339	1	0.560
Years of experience (16–20 years)	≥20 years	-0.027	0.109	0.063	1	0.802
Diploma	Doctoral degree	-0.201	0.1489	1.825	1	0.177
Bachelor’s degree	Doctoral degree	-0.209	0.1326	2.493	1	0.114
Master’s degree	Doctoral degree	-0.061	0.1431	0.184	1	0.668
Participation in specialised training (yes)	No	0.227	0.051	19.752	1	<0.001
Experience working in a specialist GDM clinic (yes)	No	0.145	0.046	9.902	1	0.002
Self-reported mastery of relevant knowledge (yes)	No	0.451	0.068	44.229	1	<0.001

Reference categories are shown in the table. P < 0.05 indicates statistical significance in the robust regression model.

**Table 4:** Robust regression analysis of factors associated with professional belief scores.

Independent variable	Reference group	*B*	SE	Wald χ²	df	*P*
Sex (male)	Female	0.014	0.0829	0.029	1	0.865
Age <30 years	≥40 years	-0.066	0.0741	0.803	1	0.370
Age 30–39 years	≥40 years	0.021	0.0442	0.219	1	0.639
Parental status (yes)	No	-0.040	0.0557	0.510	1	0.475
Hospital level: secondary hospitals	Tertiary level-A hospitals	-0.007	0.043	0.024	1	0.876
Hospital level: tertiary level-B hospitals	Tertiary level-A hospitals	0.069	0.066	1.097	1	0.295
Profession (nurse)	Doctor	-0.029	0.0455	0.415	1	0.519
Professional title (senior)	Other	0.106	0.0838	1.594	1	0.207
Professional title (associate senior)	Other	0.016	0.0639	0.066	1	0.797
Professional title (intermediate)	Other	-0.032	0.0437	0.548	1	0.459
Years of experience (≤5 years)	≥20 years	0.006	0.0704	0.006	1	0.937
Years of experience (6–10 years)	≥20 years	0.031	0.0690	0.200	1	0.655
Years of experience (11–15 years)	≥20 years	-0.038	0.0719	0.272	1	0.602
Years of experience (16–20 years)	≥20 years	0.074	0.0751	0.963	1	0.326
Diploma	Doctoral degree	-0.069	0.0889	0.598	1	0.439
Bachelor’s degree	Doctoral degree	-0.082	0.0723	1.290	1	0.256
Master’s degree	Doctoral degree	-0.066	0.0781	0.708	1	0.400
Participation in specialised training (yes)	No	0.081	0.0394	4.200	1	0.040
Experience working in a specialist GDM clinic (yes)	No	-0.009	0.0365	0.058	1	0.810
Self-reported mastery of relevant knowledge (yes)	No	0.190	0.0491	14.988	1	<0.001

Reference categories are shown in the table. P < 0.05 indicates statistical significance in the robust regression model.

**Table 5:** Robust regression analysis of factors associated with practice scores.

Independent variable	Reference group	*B*	SE	Wald χ²	df	*P*
Sex (male)	Female	0.065	0.1439	0.204	1	0.652
Age <30 years	≥40 years	0.187	0.1309	2.032	1	0.154
Age 30–39 years	≥40 years	0.084	0.0799	1.097	1	0.295
Parental status (yes)	No	0.029	0.0948	0.094	1	0.759
Hospital level: secondary hospitals	Tertiary level-A hospitals	-0.028	0.074	0.149	1	0.700
Hospital level: tertiary level-B hospitals	Tertiary level-A hospitals	0.341	0.088	15.094	1	<0.001
Profession (nurse)	Doctor	-0.030	0.0728	0.167	1	0.683
Professional title (senior)	Other	0.040	0.1551	0.068	1	0.795
Professional title (associate senior)	Other	-0.084	0.1115	0.572	1	0.450
Professional title (intermediate)	Other	-0.109	0.0810	1.829	1	0.176
Years of experience (≤5 years)	≥20 years	-0.266	0.110	5.817	1	0.016
Years of experience (6–10 years)	≥20 years	-0.210	0.1082	3.758	1	0.053
Years of experience (11–15 years)	≥20 years	-0.110	0.1106	0.991	1	0.320
Years of experience (16–20 years)	≥20 years	0.029	0.1227	0.057	1	0.811
Diploma	Doctoral degree	-0.326	0.2769	1.389	1	0.239
Bachelor’s degree	Doctoral degree	-0.244	0.2618	0.872	1	0.350
Master’s degree	Doctoral degree	-0.195	0.2642	0.547	1	0.460
Participation in specialised training (yes)	No	0.182	0.0736	6.114	1	0.013
Experience working in a specialist GDM clinic (yes)	No	0.246	0.0585	17.630	1	<0.001
Self-reported mastery of relevant knowledge (yes)	No	0.350	0.0992	12.492	1	<0.001

Reference categories are shown in the table. P < 0.05 indicates statistical significance in the robust regression model.

**Table 6:** Training, clinic experience, and knowledge mastery as predictors of obstetric healthcare professionals’ knowledge, professional beliefs, and practice.

Variable	Knowledge B (95% CI)	*P*	Professional belief B (95% CI)	*P*	Practice B (95% CI)	*P*
Training experience	0.237 (0.139–0.334)	<0.001	0.079 (0.002–0.156)	0.044	0.189 (0.044–0.333)	0.010
Clinic experience	0.133 (0.048–0.217)	0.002	0.007 (-0.058–0.072)	0.831	0.235 (0.123–0.346)	<0.001
Knowledge mastery	0.481 (0.349–0.613)	<0.001	0.185 (0.089–0.282)	<0.001	0.382 (0.187–0.577)	<0.001

**Table 6** presents parsimonious robust regression models focusing on three potentially modifiable provider-level factors. Models were fitted using generalised linear models with a normal distribution and identity link function, with robust standard errors estimated using a robust covariance matrix. The reference category for each variable was “no”.

#### 3.6.1 Knowledge dimension

Participation in specialised training (*B* = 0.227, *P* < 0.001), experience working in a specialist GDM clinic (*B* = 0.145, *P* = 0.002), and self-reported mastery of relevant knowledge (*B* = 0.451, *P* < 0.001) were significantly associated with higher knowledge scores. No statistically significant associations were observed for sex, age, parental status, hospital level, profession, professional title, years of experience, or educational level after adjustment (**Table 3**).

#### 3.6.2 Professional belief dimension

Participation in specialised training (B = 0.081, P = 0.040) and self-reported mastery of relevant knowledge (B = 0.190, P < 0.001) were positively associated with professional belief scores. After secondary level-A and secondary level-B hospitals were combined into a single secondary-hospital category, hospital level was not significantly associated with professional belief scores. Specialist GDM clinic experience and the remaining demographic variables were not statistically significant (**Table 4**).

#### 3.6.3 Practice dimension

Practice scores were significantly associated with participation in specialised training (B = 0.182, P = 0.013), experience working in a specialist GDM clinic (B = 0.246, P < 0.001), and self-reported mastery of relevant knowledge (B = 0.350, P < 0.001). In addition, hospital level was significantly associated with practice scores after secondary level-A and secondary level-B hospitals were combined into a single secondary-hospital category. Compared with tertiary level-A hospitals, secondary hospitals did not differ significantly in practice scores (B = -0.028, P = 0.700), whereas tertiary level-B hospitals had higher practice scores (B = 0.341, P < 0.001). Clinicians with ≤5 years of work experience had lower practice scores than those with ≥20 years of experience (B = -0.266, P = 0.016). Details are shown in **Table 5**.

Results from the parsimonious robust regression models showed that specialised GDM training (*B* = 0.237, 95% CI 0.139–0.334, *P* < 0.001), specialist GDM clinic experience (*B* = 0.133, 95% CI 0.048–0.217, *P* = 0.002), and self-reported mastery of relevant knowledge (*B* = 0.481, 95% CI 0.349–0.613, *P* < 0.001) were significant predictors of knowledge scores.

In the professional belief model, specialised GDM training (*B* = 0.079, 95% CI 0.002–0.156, *P* = 0.044) and self-reported mastery of relevant knowledge (*B* = 0.185, 95% CI 0.089–0.282, *P* < 0.001) were significant factors, whereas specialist GDM clinic experience was not statistically significant (*P* = 0.831).

In the practice model, specialised GDM training (*B* = 0.189, 95% CI 0.044–0.333, *P* = 0.010), specialist GDM clinic experience (*B* = 0.235, 95% CI 0.123–0.346, *P* < 0.001), and self-reported mastery of relevant knowledge (*B* = 0.382, 95% CI 0.187–0.577, *P* < 0.001) were all significantly associated with practice scores. Details are shown in **Table 6**.

### 3.7 Sensitivity analyses

Sensitivity analyses using linear mixed-effects models with province as a random intercept showed that the main findings were materially unchanged. The estimated province-level ICCs for knowledge, professional belief, and practice scores were 0.030, 0.024, and 0.038, respectively, indicating limited clustering at the provincial level. After accounting for province-level clustering, the directions and statistical significance of the key associations, particularly those for specialised GDM training, specialist GDM clinic experience, and self-reported knowledge mastery, were consistent with the robust generalised linear models. Details are presented in **Supplementary Table 6**.

## 4 Discussion

### 4.1 Provider- and institution-related gaps in postpartum glucose follow-up after GDM

The present study identified a gap between relatively high knowledge and professional belief scores and comparatively weaker implementation-related practice regarding postpartum glucose follow-up after GDM among obstetric healthcare professionals. Because all KAP items were scored on a five-point Likert scale, dimension-level mean scores ranged theoretically from 1 to 5. In this study, a mean score of 4.0 or above, corresponding to 80% of the maximum possible score and broadly reflecting “agree/often” responses, was used as a descriptive threshold for a favourable level. The mean knowledge score was 4.07 ± 0.57, which exceeded this threshold; the mean professional belief score was 3.99 ± 0.40, which was close to the threshold; and the mean practice score was 3.89 ± 0.73, which was lower than both knowledge and professional belief scores. Therefore, the findings should be interpreted as indicating relatively high knowledge, broadly positive professional beliefs, and comparatively weaker implementation-related practice, rather than uniformly favourable KAP performance.

At the item level, the knowledge dimension showed high agreement for understanding postnatal glycaemic targets and hypoglycaemia management, whereas lower agreement was observed for knowledge related to digital health technologies and the latest research evidence and clinical guidelines. In the professional belief dimension, agreement exceeded 96% for positively worded items, but many participants also agreed that postpartum glucose management increased workload and that policy support was insufficient. In contrast, implementation-related practice remained less consistent: fewer than half of participants reported that they often or always implemented postpartum glucose management and follow-up for women with GDM, and 57.21% reported tracking 4–12-week postpartum OGTT results. According to international clinical guidelines, women with GDM should undergo OGTT screening at 4–12 weeks postpartum (3, 4, 10). These findings indicate that implementation remains inconsistent, particularly with regard to follow-up processes and recommended postpartum screening.

This pattern is consistent with the Knowledge–Attitude–Practice framework, which suggests that knowledge is an important basis for behaviour but does not necessarily translate directly into practice (16, 17). The findings also suggest that limited institutional support, competing clinical priorities, and care delivery constraints may restrict implementation. Previous studies have similarly reported role ambiguity, poor communication, and difficulties in handover across different levels of healthcare institutions in GDM care (23, 24). During postpartum visits, clinicians often need to address multiple competing priorities, including assessment of postpartum haemorrhage, contraceptive counselling, and depression screening, leaving limited time for long-term metabolic risk education and follow-up management (23, 24). In addition, women in the postpartum period may prioritise newborn care and may have limited awareness of the importance of postpartum glucose follow-up, which may further hinder implementation (24, 25).

These findings suggest that improving postpartum glucose follow-up after GDM requires more than increasing clinicians’ knowledge alone. Future efforts may benefit from optimising follow-up pathways, clarifying management responsibilities across services, and strengthening institutional support for postpartum risk monitoring (24, 26). Digital health tools, such as online education and SMS reminder platforms, could be explored as potential approaches to improve awareness and follow-up engagement among women after GDM (27). Greater recognition of the postpartum period as a key stage for diabetes prevention after GDM may help reduce missed opportunities for long-term metabolic risk reduction.

Although the questionnaire was developed according to the three-domain KAP framework, exploratory factor analysis extracted four empirical factors rather than exactly reproducing the original three-dimensional structure. This finding does not indicate that the practice dimension split into two separate factors. Instead, the four-factor solution largely mapped onto the original KAP framework as follows: Factor 1 included all knowledge items; Factor 2 mainly included practice items, together with one capability-related professional belief item assessing whether clinicians could independently complete postpartum glucose management and follow-up; Factor 3 included positive professional belief items; and Factor 4 included two negatively worded professional belief items reflecting perceived workload and insufficient policy support. Therefore, the main discrepancy was that the professional belief domain separated into positive professional beliefs and perceived implementation barriers, while one capability-related professional belief item loaded with the practice factor. This pattern is clinically meaningful because willingness, perceived importance, and professional value may differ from perceived feasibility or system-level barriers in shaping implementation-related practice. In particular, clinicians may recognise the importance of postpartum glucose follow-up but still experience workload pressure, limited policy support, or insufficient institutional pathways that constrain implementation. Therefore, the four-factor solution suggests that future KAP-based assessments should distinguish between positive professional beliefs and perceived implementation barriers when evaluating postpartum glucose follow-up after GDM.

### 4.2 Role of specialised training and clinical experience in postpartum glucose follow-up after GDM

Robust regression analysis showed that specialised GDM training, prior experience in a specialist GDM clinic, and self-reported mastery of relevant knowledge were associated with better performance across the knowledge, professional belief, and practice dimensions. Correlation analysis also showed positive associations among knowledge, professional beliefs, and practice, with the strongest correlation observed between knowledge and practice. However, these findings should be interpreted cautiously because the cross-sectional design does not allow causal inference or determination of temporal direction.

Clinicians with higher self-reported knowledge mastery tended to report better implementation-related practice, but this association may have several explanations. Greater knowledge may be one component of implementation readiness; alternatively, clinicians who are already more engaged in postpartum glucose follow-up may acquire greater knowledge through routine practice. It is also possible that unmeasured factors, such as professional motivation, institutional culture, or better-organised GDM services, contributed to both higher knowledge scores and better reported practice. Therefore, knowledge mastery should be interpreted as an associated factor rather than as a demonstrated cause of better implementation.

The associations between specialised training, specialist GDM clinic experience, and KAP scores should be interpreted in a similarly cautious manner. In this study, 26.6% of participants had not received specialised training in GDM management, and 63.8% had not worked in a specialist GDM clinic. Participants with these forms of training or specialist clinical exposure reported higher scores in several domains. These associations may reflect greater familiarity with guidelines, exposure to structured care pathways, and opportunities to observe or participate in postpartum follow-up. However, reverse directionality and residual confounding cannot be excluded. For example, clinicians who are more motivated or who work in institutions with stronger GDM management systems may be more likely to receive training, work in specialist clinics, and report better follow-up practices.

These findings suggest that guideline-based education, case discussion, and exposure to specialist or interdisciplinary GDM services may be important components to consider in multifaceted strategies for improving postpartum glucose follow-up after GDM (17, 26). Nevertheless, the effectiveness of such strategies cannot be confirmed from the present cross-sectional data. Future longitudinal or intervention studies are needed to determine whether structured training, specialist clinical exposure, or knowledge-support interventions can improve implementation-related practice and postpartum follow-up outcomes.

It should be noted that the present study did not collect data from postpartum women regarding cultural beliefs, postpartum practices, or follow-up attendance; therefore, cultural factors cannot be used to explain the practice gaps observed in this sample. Nevertheless, cultural and behavioural contexts may be relevant considerations for future research on postpartum glucose follow-up after GDM. Previous studies have suggested that, after childbirth, some women may shift their attention to infant care and may perceive glycaemic problems as resolved once pregnancy has ended (24, 25). In China and some other cultural settings, postpartum recovery practices such as zuo yuezi remain common and may shape women’s diet, activity, priorities, and engagement with healthcare services (28, 29). These factors should therefore be investigated directly in future studies involving postpartum women. Such research could help clarify how cultural beliefs, family support, postpartum recovery practices, and healthcare accessibility interact with clinician counselling and institutional follow-up pathways.

### 4.3 Influence of institutional context and clinical experience on postpartum glucose follow-up after GDM

In this study, implementation-related practice scores differed across hospital levels and years of experience. Participants were mainly from tertiary level-A hospitals, followed by secondary level-A hospitals, tertiary level-B hospitals, and secondary level-B hospitals. Because only two participants were recruited from secondary level-B hospitals, secondary level-A and secondary level-B hospitals were combined into a single secondary-hospital category before the primary multivariable analyses. In the revised primary analysis, secondary hospitals did not differ significantly from tertiary level-A hospitals, whereas tertiary level-B hospitals reported higher practice scores. Therefore, hospital-level differences should not be interpreted as a simple gradient according to hospital level.

Several contextual factors may help explain these observed associations. Some lower-level or less well-resourced healthcare institutions may have fewer staff, less developed follow-up systems, and weaker coordination across departments and services (23, 24). Such institutional conditions may be associated with less consistent implementation of postpartum glucose follow-up. In the present study, many respondents also agreed with negatively worded items indicating increased workload and insufficient policy support, which further suggests that system-level pressures may be relevant to implementation gaps in routine practice (23, 24). However, because residual hospital-level clustering cannot be fully excluded, these interpretations should be considered hypothesis-generating and require confirmation in future studies using clearly defined hospital identifiers or multilevel sampling designs.

The association between fewer years of work experience and lower practice scores may also have multiple explanations. Early-career clinicians may have less familiarity with postpartum follow-up procedures, less confidence in long-term risk communication, or fewer opportunities to coordinate care across specialties. However, this association may also reflect unmeasured contextual factors, such as differences in role responsibilities, training opportunities, workload, or institutional support. Therefore, years of experience should be understood as an associated professional characteristic rather than as a causal determinant of practice.

Taken together, these findings suggest that capacity-building efforts may need to consider both professional and institutional contexts. Standardised, practice-oriented training for early-career clinicians, locally appropriate postpartum follow-up pathways, and experience-sharing across institutions could be considered as possible components of service improvement (17, 26). However, whether these approaches can improve postpartum glucose follow-up after GDM requires evaluation in future longitudinal or intervention-based studies.

This issue may be particularly relevant in health systems where resources are unevenly distributed, including China and many other developing settings. Strengthening the capacity of lower-level institutions to support postpartum glucose follow-up after GDM could potentially contribute to more consistent care, but the present findings should be interpreted as hypothesis-generating rather than causal evidence.

### 4.4 Strengths and limitations

This study has several strengths. First, it focused on postpartum glucose follow-up after GDM, an area for which evidence from routine obstetric practice remains limited. Second, the questionnaire was developed within a Knowledge–Attitude–Practice framework and refined through two rounds of Delphi expert consultation, which helped improve its content relevance. Third, this study included obstetric healthcare professionals from 128 participating urban public hospitals across 15 provinces in China, providing a broad descriptive snapshot of postpartum glucose follow-up practices across multiple urban public hospital settings and institutional contexts. However, this geographic breadth should not be interpreted as eliminating selection bias or as providing nationally representative estimates.

Several limitations should also be acknowledged. First, because of the cross-sectional design, causal relationships among the variables cannot be established. In particular, the findings do not demonstrate that specialised GDM training, specialist GDM clinic experience, or self-reported knowledge mastery caused higher knowledge, professional belief, or practice scores. Reverse directionality is possible, as healthcare professionals who are already more engaged in postpartum glucose follow-up may be more likely to seek training, work in specialist GDM services, or report higher knowledge mastery. In addition, unmeasured factors, such as professional motivation, institutional culture, staffing, workload, or established GDM care pathways, may have influenced both exposure to training or specialist services and implementation-related practice. Therefore, the observed associations should be interpreted as exploratory and hypothesis-generating.

Second, this study used convenience sampling to recruit hospitals and participants, which may have introduced substantial selection bias. Although the sample included obstetric healthcare professionals from 128 participating public hospitals across 15 provinces, all participating hospitals were located in urban areas and were not randomly selected. Therefore, the findings should be interpreted as a broad descriptive overview of postpartum glucose follow-up practice among participating urban public hospitals rather than as nationally representative estimates. In addition, because the survey was distributed through local institutional contacts and completed anonymously, information on non-respondents was unavailable, and we could not compare respondents with non-respondents or with national workforce demographic data. Self-selection may have led to over-representation of healthcare professionals or institutions with greater interest in GDM management and postpartum follow-up, which may have biased estimates of knowledge, professional beliefs, and implementation-related practice upward. Thus, the relatively high knowledge and professional belief scores observed in this study may not reflect the situation in all obstetric healthcare settings in China, particularly in rural, private, primary-care, or less-resourced institutions.

Third, hospital-level clustering could not be adequately modelled. This represents a major limitation of the present study. Healthcare professionals were recruited from 128 hospitals, and responses from participants working in the same hospital may be correlated. However, specific hospital identifiers could not be reliably defined in the de-identified analytic dataset; therefore, hospital-level random intercepts or hospital-clustered standard errors could not be specified. Although province was included as a random intercept in sensitivity analyses, province-level modelling cannot substitute for direct modelling of clustering within hospitals. Hospital-level intraclass correlation may materially affect inferential validity, particularly for institution-related variables such as hospital level. Therefore, hospital-level findings should be interpreted cautiously, and future studies should use clearly defined hospital identifiers or multilevel sampling designs to allow direct modelling of hospital-level clustering.

Fourth, the distribution of participants across hospital levels was uneven. Because only two participants were recruited from secondary level-B hospitals, secondary level-A and secondary level-B hospitals were combined into a single secondary-hospital category before the primary multivariable analyses to improve the stability of hospital-level estimates. This approach reduced the influence of an extremely small subgroup and improved statistical stability, but it also reduced the granularity of hospital-level comparisons and may have masked differences between secondary level-A and secondary level-B hospitals. Moreover, the number of participants from non-tertiary hospitals remained limited. Therefore, institution-related findings should not be interpreted as demonstrating a simple hospital-level gradient and require confirmation in future studies with adequate sample sizes across hospital categories.

Fifth, the data were based primarily on self-report and may therefore have been affected by social desirability bias. Healthcare professionals may have overestimated their knowledge, professional beliefs, or implementation-related practice regarding postpartum glucose follow-up after GDM.

Finally, although the questionnaire underwent expert consultation and exploratory factor analysis, EFA extracted four empirical factors rather than exactly reproducing the original three-domain KAP structure. The professional belief component separated into positive professional beliefs and perceived implementation barriers, and one capability-related belief item loaded with the practice factor. This suggests that the professional belief component of postpartum glucose follow-up after GDM may be more complex than originally assumed in the three-domain KAP framework. In addition, the high Cronbach’s α values for some dimensions may suggest possible overlap among items; therefore, further psychometric validation, including confirmatory factor analysis in independent samples, is warranted.

Future studies could build on these findings using longitudinal, multicentre, or intervention-based designs with probability-based or stratified sampling, adequate representation of different hospital levels, and clearly defined hospital identifiers. Such studies would help clarify how provider-level factors, institutional context, and hospital-level clustering influence implementation of postpartum glucose follow-up after GDM in routine care.

## 5 Conclusions

This study suggests that implementation of postpartum glucose follow-up after GDM in routine obstetric care is associated with both provider- and institution-related factors. Although obstetric healthcare professionals generally demonstrated good knowledge and positive professional beliefs, practice remained comparatively weaker. Specialised training, clinical experience, and institutional context were associated with implementation-related practice. Future efforts to strengthen training, optimise follow-up pathways, and improve institutional support may help reduce missed opportunities for postpartum follow-up and strengthen long-term diabetes prevention after GDM.

## Data Availability

The original contributions presented in the study are included in the article/**Supplementary Material**. Further inquiries can be directed to the corresponding author.
