# Supplementary material for: Postpartum glucose follow-up after gestational diabetes in China: provider and institutional determinants in a multicenter survey across urban public hospitals
Source: Front Endocrinol (Lausanne). 2026 Aug 7;17:1887259. doi: 10.3389/fendo.2026.1887259 (PMC13493272; doi:10.3389/fendo.2026.1887259)
Supplement: Supplementary file 1 [file Table1.docx]

This file includes: Supplementary Table 1. Item-level content validity index (I-CVI) for the questionnaire items (*n* = 17).

| Item | Number of experts | Number rating 3 or 4 | I-CVI | Item | Number of experts | Number rating 3 or 4 | I-CVI |
| --- | --- | --- | --- | --- | --- | --- | --- |
| K1 | 17 | 16 | 0.941 | A6 | 17 | 17 | 1.000 |
| K2 | 17 | 16 | 0.941 | A7 | 17 | 16 | 0.941 |
| K3 | 17 | 16 | 0.941 | A8 | 17 | 17 | 1.000 |
| K4 | 17 | 16 | 0.941 | A9 | 17 | 15 | 0.882 |
| K5 | 17 | 17 | 1.000 | A10 | 17 | 15 | 0.882 |
| K6 | 17 | 17 | 1.000 | P1 | 17 | 14 | 0.823 |
| K7 | 17 | 17 | 1.000 | P2 | 17 | 16 | 0.941 |
| K8 | 17 | 17 | 1.000 | P3 | 17 | 17 | 1.000 |
| K9 | 17 | 17 | 1.000 | P4 | 17 | 17 | 1.000 |
| K10 | 17 | 17 | 1.000 | P5 | 17 | 17 | 1.000 |
| K11 | 17 | 17 | 1.000 | P6 | 17 | 17 | 1.000 |
| K12 | 17 | 17 | 1.000 | P7 | 17 | 17 | 1.000 |
| K13 | 17 | 14 | 0.824 | P8 | 17 | 17 | 1.000 |
| K14 | 17 | 16 | 0.941 | P9 | 17 | 17 | 1.000 |
| K15 | 17 | 17 | 1.000 | P10 | 17 | 16 | 0.941 |
| K16 | 17 | 17 | 1.000 | P11 | 17 | 15 | 0.882 |
| A1 | 17 | 16 | 0.941 | P12 | 17 | 14 | 0.824 |
| A2 | 17 | 17 | 1.000 | P13 | 17 | 16 | 0.941 |
| A3 | 17 | 17 | 1.000 | P14 | 17 | 14 | 0.824 |
| A4 | 17 | 17 | 1.000 | P15 | 17 | 15 | 0.882 |
| A5 | 17 | 17 | 1.000 |  |  |  |  |

Supplementary Table 2. General characteristics of participants and their affiliated hospitals (n = 638).

| Variable | Category | n | % |
| --- | --- | --- | --- |
| Sex | Male | 15 | 2.4 |
|  | Female | 623 | 97.6 |
| Ethnicity | Han | 591 | 92.6 |
|  | Ethnic minority | 47 | 7.4 |
| Parental status | Yes | 513 | 80.4 |
|  | No | 125 | 19.6 |
| Hospital level | Secondary hospitals | 104 | 16.3 |
|  | Tertiary level-B | 39 | 6.1 |
|  | Tertiary level-A | 495 | 77.6 |
| Profession | Nurse | 496 | 77.7 |
|  | Doctor | 142 | 22.3 |
| Professional title | Senior title | 28 | 4.4 |
|  | Associate senior title | 101 | 15.8 |
|  | Intermediate title | 322 | 50.5 |
|  | Other | 187 | 29.3 |
| Years of experience in managing women with GDM | ≤5 years | 239 | 37.5 |
|  | 6–10 years | 176 | 27.6 |
|  | 11–15 years | 112 | 17.6 |
|  | 16–20 years | 60 | 9.4 |
|  | ≥20 years | 51 | 8.0 |
| Highest educational qualification | Diploma | 79 | 12.4 |
|  | Bachelor’s degree | 502 | 78.7 |
|  | Master’s degree | 48 | 7.5 |
|  | Doctoral degree | 9 | 1.4 |
| Participation in specialised GDM management training | Yes | 468 | 73.4 |
|  | No | 170 | 26.6 |
| Experience working in a specialist GDM clinic | Yes | 231 | 36.2 |
|  | No | 407 | 63.8 |
| Self-reported mastery of knowledge related to postpartum glucose management after GDM | Yes | 550 | 86.2 |
|  | No | 88 | 13.8 |

Supplementary Table 3. Item scores and agreement rates for obstetric healthcare professionals’ KAP regarding postpartum glucose follow-up after GDM (N = 638).

| Item | Mean ± SD | Agreement rate (%) |
| --- | --- | --- |
| K1 | 4.18 ± 0.66 | 92.63 |
| K2 | 4.14 ± 0.67 | 89.97 |
| K3 | 4.03 ± 0.74 | 84.64 |
| K4 | 4.03 ± 0.73 | 84.01 |
| K5 | 4.05 ± 0.71 | 85.89 |
| K6 | 3.91 ± 0.78 | 77.74 |
| K7 | 4.12 ± 0.64 | 90.60 |
| K8 | 4.22 ± 0.62 | 94.20 |
| K9 | 4.24 ± 0.62 | 94.04 |
| K10 | 4.07 ± 0.69 | 86.68 |
| K11 | 4.09 ± 0.67 | 88.09 |
| K12 | 4.13 ± 0.65 | 90.60 |
| K13 | 4.10 ± 0.69 | 88.40 |
| K14 | 3.85 ± 0.86 | 72.88 |
| K15 | 3.89 ± 0.81 | 76.96 |
| K16 | 4.08 ± 0.66 | 87.46 |
| A1 | 4.54 ± 0.57 | 97.65 |
| A2 | 4.45 ± 0.58 | 96.87 |
| A3 | 4.53 ± 0.55 | 98.28 |
| A4 | 4.52 ± 0.56 | 97.49 |
| A5 | 4.47 ± 0.57 | 96.39 |
| A6 | 4.52 ± 0.54 | 98.43 |
| A7 | 4.14 ± 0.78 | 81.19 |
| A8 | 4.41 ± 0.57 | 96.87 |
| A9 (reverse item) | 3.87 ± 1.13 | 76.65 |
| A10 (reverse item) | 3.82 ± 1.04 | 71.32 |
| P1 | 3.44 ± 1.12 | 49.84 |
| P2 | 3.76 ± 0.91 | 63.64 |
| P3 | 4.00 ± 0.88 | 77.27 |
| P4 | 3.93 ± 0.94 | 74.29 |
| P5 | 3.81 ± 1.02 | 68.50 |
| P6 | 3.55 ± 1.20 | 57.21 |
| P7 | 3.93 ± 1.05 | 74.14 |
| P8 | 4.07 ± 0.84 | 80.09 |
| P9 | 4.29 ± 0.75 | 89.03 |
| P10 | 4.10 ± 0.89 | 80.25 |
| P11 | 3.82 ± 1.07 | 68.97 |
| P12 | 3.83 ± 0.99 | 67.40 |
| P13 | 3.98 ± 0.92 | 75.08 |
| P14 | 3.75 ± 1.07 | 64.11 |
| P15 | 4.09 ± 0.89 | 78.53 |

**Note:** Agreement rate was defined as “know/know very well” for knowledge items, “agree/strongly agree” for professional belief items, and “often/always” for practice items.

Supplementary Table 4. Differences in knowledge, professional belief, and practice scores according to participant characteristics (*N* = 638)

| Variable | Group | N | Knowledge (Mean ± SD) | Professional belief (Mean ± SD) | Practice (Mean ± SD) | Statistic | *P* |
| --- | --- | --- | --- | --- | --- | --- | --- |
| Sex | Male | 15 | 4.24 ± 0.46 | 4.09 ± 0.33 | 4.06 ± 0.70 | *Z* = -1.422 | 0.155 |
|  | Female | 623 | 4.01 ± 0.57 | 3.99 ± 0.40 | 3.88 ± 0.73 |  |  |
| Participation in specialised training | Yes | 468 | 4.19 ± 0.52 | 4.02 ± 0.38 | 3.99 ± 0.68 | *Z* = -8.497 | <0.001 |
|  | No | 170 | 3.75 ± 0.59 | 3.89 ± 0.42 | 3.60 ± 0.79 |  |  |
| Experience working in a specialist GDM clinic | Yes | 231 | 4.26 ± 0.54 | 4.03 ± 0.37 | 4.12 ± 0.60 | *Z* = -6.045 | <0.001 |
|  | No | 407 | 3.97 ± 0.56 | 3.96 ± 0.41 | 3.76 ± 0.77 |  |  |
| Self-reported mastery of relevant knowledge | Yes | 550 | 4.16 ± 0.51 | 4.02 ± 0.39 | 3.97 ± 0.68 | *Z* = -9.350 | <0.001 |
|  | No | 88 | 3.51 ± 0.59 | 3.79 ± 0.41 | 3.41 ± 0.84 |  |  |
| Hospital level | Secondary hospitals | 104 | 3.99 ± 0.52 | 3.97 ± 0.39 | 3.82 ± 0.78 | Knowledge: χ² = 8.236 | 0.016 |
|  | Tertiary level-B | 39 | 4.29 ± 0.50 | 4.07 ± 0.40 | 4.28 ± 0.53 | Professional beliefs: χ² = 2.935 | 0.230 |
|  | Tertiary level-A | 495 | 4.07 ± 0.58 | 3.98 ± 0.40 | 3.87 ± 0.72 | Practice: χ² = 12.862 | 0.002 |
| Years of experience | ≤5 years | 239 | 3.97 ± 0.59 | 3.96 ± 0.41 | 3.80 ± 0.76 | χ² = 10.292 | 0.036 |
|  | 6–10 years | 176 | 4.14 ± 0.53 | 4.02 ± 0.39 | 3.88 ± 0.69 |  |  |
|  | 11–15 years | 112 | 4.10 ± 0.49 | 3.96 ± 0.38 | 3.94 ± 0.76 |  |  |
|  | 16–20 years | 60 | 4.15 ± 0.70 | 4.07 ± 0.38 | 4.10 ± 0.74 |  |  |
|  | ≥20 years | 51 | 4.16 ± 0.55 | 3.99 ± 0.43 | 3.99 ± 0.58 |  |  |
|  |  |  |  |  |  |  |  |

Note: For hospital level, secondary level-A and secondary level-B hospitals were combined into a single secondary-hospital category. Kruskal–Wallis H tests showed significant differences in knowledge scores (χ² = 8.236, df = 2, P = 0.016) and practice scores (χ² = 12.862, df = 2, P = 0.002), but not in professional belief scores (χ² = 2.935, df = 2, P = 0.230). Post hoc pairwise comparisons with Bonferroni correction showed that knowledge scores were higher in tertiary level-B hospitals than in secondary hospitals (adjusted P = 0.012). Practice scores were higher in tertiary level-B hospitals than in secondary hospitals (adjusted P = 0.003) and tertiary level-A hospitals (adjusted P = 0.001). For years of experience, Kruskal–Wallis H tests showed significant differences in knowledge scores (χ² = 10.292, df = 4, P = 0.036) and practice scores (χ² = 10.319, df = 4, P = 0.035), but not in professional belief scores (χ² = 5.597, df = 4, P = 0.231).

Supplementary Table 5. Summary of the rotated factor loading matrix for the KAP questionnaire.

| Factor | Interpretation | Items primarily loading on the factor | Primary loading range | Notes |
| --- | --- | --- | --- | --- |
| Factor 1 | Knowledge | K1–K16 | 0.628–0.812 | All knowledge items loaded on this factor |
| Factor 2 | Practice / implementation-related behaviours | P1–P15 and A7 | P items: 0.505–0.793; A7: 0.465 | A7 assessed perceived capability to independently perform postpartum glucose management and follow-up |
| Factor 3 | Positive professional beliefs | A1–A6 and A8 | 0.690–0.877 | Positive professional belief, interest, willingness, perceived importance, and professional value |
| Factor 4 | Perceived implementation barriers | A9–A10 | 0.824–0.826 | Negatively worded items on workload and policy support |

Supplementary Table 6. Sensitivity analyses using linear mixed-effects models with province as a random intercept.

| Outcome | Variable | B | 95% CI | P |
| --- | --- | --- | --- | --- |
| Knowledge | Specialised GDM training | 0.227 | 0.124–0.331 | <0.001 |
| Knowledge | Specialist GDM clinic experience | 0.139 | 0.045–0.233 | 0.004 |
| Knowledge | Self-reported knowledge mastery | 0.450 | 0.323–0.578 | <0.001 |
| Professional belief | Specialised GDM training | 0.080 | 0.001–0.159 | 0.047 |
| Professional belief | Specialist GDM clinic experience | -0.005 | -0.077–0.066 | 0.880 |
| Professional belief | Self-reported knowledge mastery | 0.203 | 0.106–0.301 | <0.001 |
| Practice | Specialised GDM training | 0.180 | 0.040–0.319 | 0.012 |
| Practice | Specialist GDM clinic experience | 0.239 | 0.112–0.365 | <0.001 |
| Practice | Self-reported knowledge mastery | 0.354 | 0.182–0.525 | <0.001 |

Note: Linear mixed-effects models included province as a random intercept and the same prespecified fixed-effect variables as the full multivariable models. The table presents key provider-level variables. The reference category for each variable was “no”. Province-level ICCs for knowledge, professional belief, and practice scores were 0.030, 0.024, and 0.038, respectively.

Supplementary Table 7. Multicollinearity diagnostics for predictors included in the multivariable models

| Predictor | Reference category | Tolerance | VIF |
| --- | --- | --- | --- |
| Sex: male | Female | 0.868 | 1.152 |
| Age <30 years | ≥40 years | 0.316 | 3.166 |
| Age 30–39 years | ≥40 years | 0.452 | 2.213 |
| Parental status: yes | No | 0.540 | 1.852 |
| Profession: nurse | Doctor | 0.630 | 1.586 |
| Professional title: senior | Other | 0.663 | 1.508 |
| Professional title: associate senior | Other | 0.428 | 2.335 |
| Professional title: intermediate | Other | 0.494 | 2.025 |
| Years of experience: ≤5 years | ≥20 years | 0.205 | 4.888 |
| Years of experience: 6–10 years | ≥20 years | 0.243 | 4.118 |
| Years of experience: 11–15 years | ≥20 years | 0.323 | 3.093 |
| Years of experience: 16–20 years | ≥20 years | 0.474 | 2.111 |
| Educational qualification: diploma | Bachelor’s degree | 0.895 | 1.118 |
| Educational qualification: master’s degree | Bachelor’s degree | 0.728 | 1.374 |
| Educational qualification: doctoral degree | Bachelor’s degree | 0.870 | 1.149 |
| Participation in specialised GDM training: yes | No | 0.744 | 1.343 |
| Experience working in a specialist GDM clinic: yes | No | 0.764 | 1.309 |
| Self-reported mastery of relevant knowledge: yes | No | 0.805 | 1.242 |
| Hospital level: secondary hospitals | Tertiary level-A hospitals | 0.908 | 1.101 |
| Hospital level: tertiary level-B hospitals | Tertiary level-A hospitals | 0.967 | 1.034 |

Note: VIF, variance inflation factor. Categorical predictors were dummy-coded for multicollinearity diagnostics. Because the doctoral degree subgroup was small, bachelor’s degree, the largest educational category, was used as the reference category for educational qualification in the diagnostic model. The maximum condition index was 20.587. No severe multicollinearity was indicated.
